# Supplementary material for: Patterns of Intron Gain and Loss in Fungi
Source: PLoS Biol. 2004 Nov 30;2(12):e422. doi: 10.1371/journal.pbio.0020422 (PMC532390; doi:10.1371/journal.pbio.0020422)
Supplement: Table S1 — Also available at http://genes.mit.edu/NielsenEtAl/. (4.3 MB ZIP). [file pbio.0020422.st001.zip › NielsenEtAl/html/1217.html]

AN8566.1.NCU04072.1.MG08983.1.FG03349.1


```
 CLUSTAL W (1.82) Multiple Sequence Alignments - Introns Inserted


Sequence 1: MG08983.1	319 aa
Sequence 2: FG03349.1	317 aa
Sequence 3: AN8566.1	313 aa
Sequence 4: NCU04072.1	297 aa
Alignment Length: 331 aa
Number Identitical Residues: 141 aa
Alignment Score (without introns) 7349


MG08983.1 	MASENTNGAT-KPAESKFDPNFTQHVIDLMSPETLPRHREILTSLIRHMHDFCREVELKQ
NCU04072.1	-----------MAS-HRFDPNFTQNVIDGMGPNTTPRNRQVLGALIRHIHDFAREVELTM
FG03349.1 	-MSNGTNGTNGAEAQSKFDPNFTDHVIGLMSPETEPRQREILTSLIRHMHDFCREVELKQ
AN8566.1  	-----------MSTNPRFDPNFTPYVINSMGPKTPERARVILGSLIRHIHDFAREVELTP
          	             :. :******  **. *.*:*  * * :* :****:***.*****. 

MG08983.1 	DEWVLGVNYINSLGQAYKK---NRNETWRLMDILGIES2LVDEINNKVVTPDGKAPTSSA
NCU04072.1	DEWMAGVKYINEVGRVYSESGQTRNEAHRLSDILGLET2LVDEIAHKIVVEEGLEPTSSS
FG03349.1 	EEWIIGVNYINSLGQAYKK---NRNETWRVCDILGIES2LVDEINHKVVTEGGKAPTSSS
AN8566.1  	AEWMLGVEFINSIGKISTP---IRNECHRICDVIGLES2LVDEIANRIVTEQGLSPTSNV
          	 **: **::**.:*:  .     ***  *: *::*:*: ***** :::*.  *  ***. 

MG08983.1 	ILGPFWSPETPFRDLGASCVQD-MPADGQLTRFHGVIRDVETGKGIPGAVFDMWQASTNG
NCU04072.1	ILGPFWSPNAPFRENGGSIIQDGVPPKGRVTKMHGVIRDITTGKPIPNAVFDIWQASANG
FG03349.1 	ILGPFWSPETPFRDLGGSVVQD-MPKDGQLTFFHGVIRDVDTGKGIPNAVFDMWQASTNG
AN8566.1  	ILGPFWSPNAPFRELGDSIIQD-PNPNGKVTFMHGVLRDMETGAPIAGAVLDIWQASANG
          	********::***: * * :**    .*::* :***:**: **  *..**:*:****:**

MG08983.1 	KYDVFDPENQTRHNLRGKFKTDEEGRFWFYCLKPTEYAIDTTGPSAVLLKMMHRHPYRPA
NCU04072.1	KYDFQDPQNQTPNNLRGKFKANEKGEYWFYCLHPTAYSLPTDGPSYALLQLMDRHPMRPA
FG03349.1 	KYDAHDPENQSRHNLRGKFRTDQDGKFWFYCLKPTEYAIDTSGPSAELLRIMGRHPYRPA
AN8566.1  	QYDFQDP-NQSENNLRGKFRSNEKGEFYWYCYHPTPYSLPTDGPAGVLLNLMDRSPMRPA
          	:**  ** **: :******::::.*.:::** :** *:: * **:  **.:* * * ***

MG08983.1 	HIHMMVTHPDYLGITAQLYPRDDPYLETDTVSAVKPDLLLDFNPAAKDDPMGAQLDCEYN
NCU04072.1	HIHIMVTHPEYQGCTTQLYPSDDPWITNDTVFAVKDDLVVTFKP-IEGDEK-AVLELEYN
FG03349.1 	HIHIMVTHDDYLGVTAQLYPSDDPYLETDTACAVKDDLVLDFKP-AKDDPKGATLDVEYN
AN8566.1  	HIHLMITHPDYATVINQIYPSDDPHLDIDSVFAVKDDLVVDFKP-KTDDPK-AELDLEYN
          	***:*:** :*     *:** *** :  *:. *** **:: *:*   .*   * *: ***

MG08983.1 	VNMVSKKYKPDS------TMLLGNANQGNFD-
NCU04072.1	VNLAPKGFK------------------GKV--
FG03349.1 	INLASKKYKPDN------TMLMQNANQDKF--
AN8566.1  	VKMALKKHHPNPNSAPPVSSFERYNKAGKEKL
          	:::. * .:.. .::.. :      . .: .
```
